# Cone Beam Computed Tomography-Based Anatomical Assessment of the Olfactory Fossa

**DOI:** 10.1155/2019/4134260

**Published:** 2019-04-01

**Authors:** Andre Luiz Ferreira Costa, Aline Kataki Paixão, Bianca Costa Gonçalves, Celso Massahiro Ogawa, Thiago Martinelli, Fernando Akio Maeda, Tarcila Trivino, Sérgio Lucio Pereira de Castro Lopes

**Affiliations:** ^1^Postgraduate Program in Dentistry, Cruzeiro do Sul University (UNICSUL), São Paulo, SP, Brazil; ^2^Department of Diagnosis and Surgery, São José dos Campos Dental School, São Paulo State University (UNESP), São José dos Campos, SP, Brazil; ^3^Department of Orthodontics and Radiology, University of São Paulo City (UNICID), São Paulo, SP, Brazil

## Abstract

This study aimed to investigate the olfactory fossa according to the Keros classification using cone beam computed tomography. This cross-sectional study analysed cone beam computed tomography images selected from a database belonging to a radiology centre. The scans of 174 healthy patients were analysed by using the Xoran software. Gender, age, and side were correlated with the Keros classification. The mean age of the 174 patients was 45.3 years. The most prevalent Keros classification was type II (65.52%), followed by type III (20.69%) and type I (13.79%). No significant differences were found between Keros classification and the variables age, right side (*p* value = 0.4620), and left side (*p* value = 0.5709). There were also no significant differences between gender and the variables right side (*p* value = 0.1421) and left side (*p* value = 0.2136). Based on these results, we suggest that cone beam computed tomography can be recommended for analysis of the anterior skull base. Keros type II was the most prevalent type in our sample.

## 1. Introduction

Endoscopic surgery of paranasal sinuses is the chosen technique for treatment of chronic rhinosinusitis and clinical management of many disorders such as mucoceles, nasal polyposis, and sellar tumours. Despite the low incidence of intraoperative complications, there may be other serious complications such as intraorbital bruises with visual loss and intracranial penetration, which are mostly caused due to manipulation of the frontal, sphenoid, and ethmoidal sinuses [1–4].

In this context, the fragility of the medial cribriform plate of the ethmoid bone, which is one of its thinnest structures, allows it to be perforated during surgical procedures due to its low resistance, resulting in the above-cited complications [2, 5, 6].

Keros in 1962 classified the olfactory fossa into three categories based on the length of the lateral lamella of the cribriform plate of the ethmoid bone (i.e., types I, II, and III) and determined the iatrogenic risk during surgical manipulations in the ethmoidal region [7].

Several studies have shown the importance of the analysis of the ethmoidal roof and its value in the prevention of endoscopic surgery complications. In agreement with the literature, iatrogenic lesions of the cranial base occur mostly in the lateral portion of the cribriform plate. Interestingly, this is the site where the ethmoidal artery penetrates the cranial fossa as it is the thinnest and least resistant part of the entire cranial base [2, 6, 8].

The multislice computed tomography (MSCT) is the gold standard in the evaluation of anatomy and diseases of paranasal sinuses and nasal cavity, allowing simultaneous visualisation of bone, soft tissue, and air [9–11]. Additionally, MSCT can reveal predispositions for the development of chronic sinusitis, trauma, and tumour based on anatomical variations and guide therapeutic endoscopic procedures [10]. However, despite all these benefits, MSCT delivers excessive ionizing radiation dose in a critical anatomic area of patients. The essential principle to guide the diagnostic use of radiation is to keep the radiation exposure as low as reasonably achievable.

Cone beam computed tomography (CBCT) has recently become an alternative method in dentistry and otorhinolaryngology for analysis of aerial spaces and paranasal sinuses as it provides higher resolution images with isometric voxel size, low radiation exposure, and lower costs [11–13].

To our knowledge, there is only one study on the accuracy of CBCT for assessment of the olfactory fossa by using Keros classification [14].

This study aims to provide comprehensive data with regard to the variations of the Keros classification in a Brazilian sample, based on CBCT scans.

## 2. Materials and Methods

This study followed the universally accepted standards for research in human beings and was approved by the Institutional Review Board of the São Paulo State University (UNESP) according to protocol number 39525814.7.0000.0077.

### 2.1. Sample Selection

Initially, 228 CBCT scans were retrospectively retrieved from the database of the Division of Dentomaxillofacial Radiology, School of Dentistry of São José dos Campos, UNESP. CBCT scans were acquired by using an i-CAT scanner (Imaging Sciences, Hatfield, PA, USA) with the following acquisition parameters: 120 kVp, 10 mA, reconstructed voxel size of 0.25 mm, and a field of view (FOV) of 8 × 16 cm. CBCT images were taken for evaluation of implant placement, impacted teeth, obstructive sleep apnea, and preorthodontic or preprosthetic treatment. There was no preference for gender regarding sample selection.

All the CBCT scans were selected according to the following inclusion/exclusion criteria:

#### 2.1.1. Inclusion Criteria

Patients older than 18 years and whose images showed the medium and superior regions of the face, so that the crista galli of the ethmoidal bone and nasal fossa could be examined, were included in the study.

#### 2.1.2. Exclusion Criteria

Patients with a history of paranasal sinus surgery, maxillofacial trauma, and pathological processes in the paranasal sinuses or with low-quality images or images containing artifacts, making visualisation of anatomical structures difficult, were excluded from the study.

From the initial sample, 47 subjects were excluded because they had images with presence of sinusopathies and 7 for presenting images with poor quality of analysis of the acquired region, from patients' movements during the acquisition. The final sample which included 174 CBCT scans of 143 females and 31 males, with ages between 18 and 86 years, were selected.

### 2.2. Image Analysis

All CBCT scans were evaluated in the XSTD format and analysed by a dentomaxillofacial radiologist with experience in CBCT imaging [15, 16] by means of Xoran software (Xoran Technologies, Ann Arbor, Michigan, USA) on a 23.8 inches LCD monitor (Dell Ultrasharp, wide screen flat-panel monitor).

**Figure 1:**
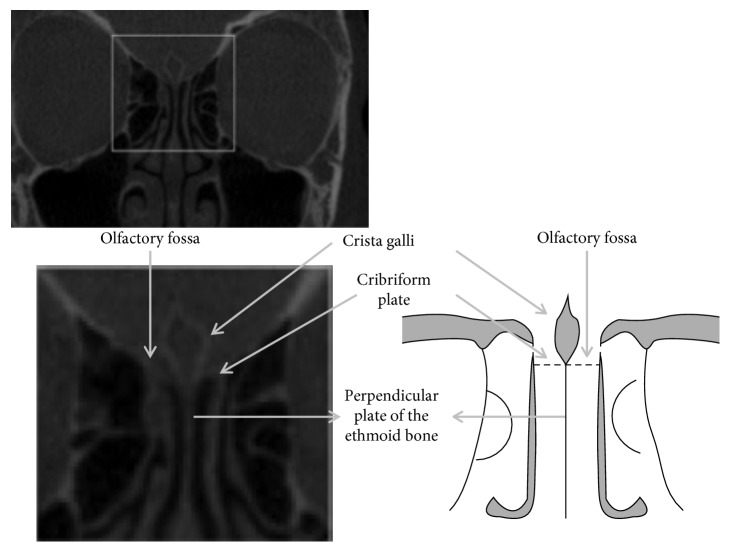
Coronal plane CBCT image and diagram with the marked ethmoidal region showing different anatomical structures.

**Figure 2:**
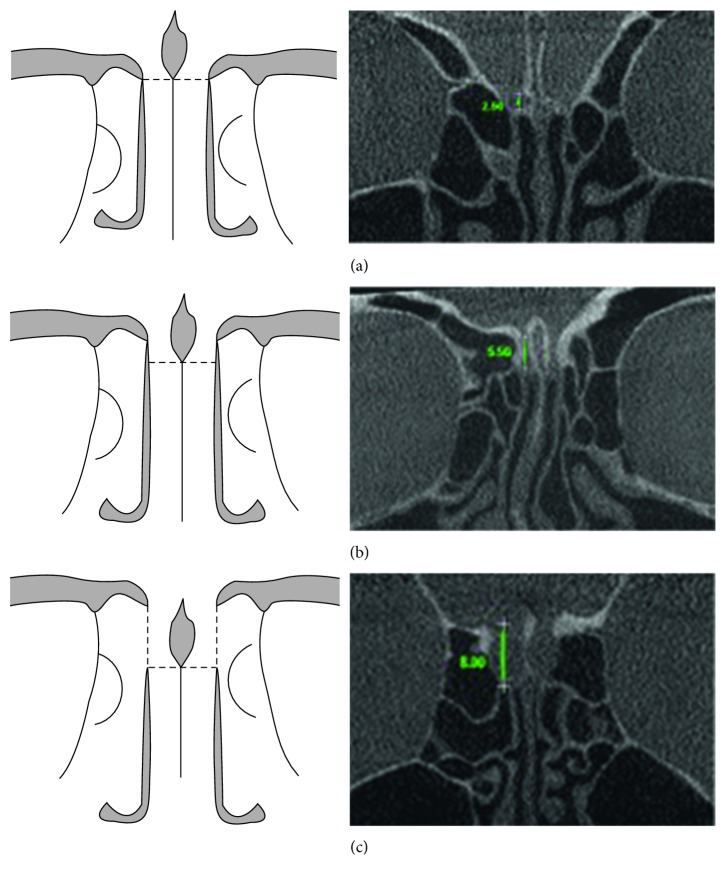
Diagram and images of measurement of the three types of olfactory fossa, according to Keros classification: (a) type I; (b) type II; (c) type III.

On the coronal sections, crista galli, perpendicular plate of the ethmoidal bone, and olfactory fossa were better visualised (Figure 1), and linear measurements (in millimeters) were made by using the software's ruler tool for both lateral lamellas of the cribriform plate (right and left sides), that is, from the lowest point of the olfactory fossa to the cribriform plate (Figure 2). The measurements were performed with the use of own software, and the resulting values were then classified into types I, II, and III for both sides (Figure 2).

The measurements obtained were used for evaluation of the olfactory fossa based on its depth, according to Keros classification [7, 17], as follows:

- Type I—height lower than 3.0 mm
- Type II—height between 4.0 and 7.0 mm
- Type III—height between 8.0 and 16.0 mm

After a 15-day interval, all the measurements were repeated for analysis of intrarater reliability.

### 2.3. Statistical Analysis

The agreement between the two assessments was verified through scatter plots, and the symmetry test was used to compare the sides in relation to the Keros classification. Gender comparison was performed by using the chi-square test for each side and between ages by using ANOVA. Generalized estimating equations (GEE) model was used to compare genders and ages for both sides as repeated measurements. All data were statistically analysed by using the SAS System for Windows (Statistical Analysis System), version 9.4 (SAS Institute Inc, Cary, NC, USA), at the significance level of 5%.

## 3. Results

**Figure 3:**
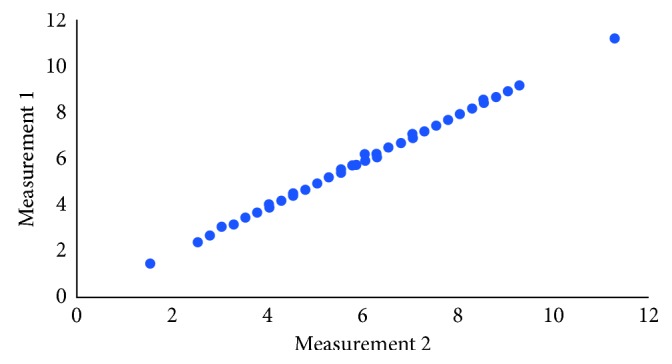
Scatter plot of the two measurements (right side).

**Figure 4:**
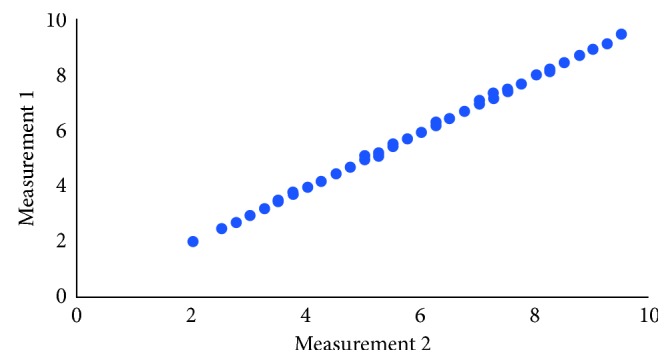
Scatter plot of the two measurements (left side).

Figures 3 and 4 show that there was an almost perfect agreement between the measurements performed at different times. Given the high correlation between the two measurements, the average of both was considered for analysis.

The ages of the patients studied ranged from 18 to 78 years old, with a mean age of 45.33 (SD = 10.7).

Table 1 shows that the Keros score was equal on both sides in 110 (63.2%) of the 174 patients, with the Kappa coefficient showing a weak agreement between the sides (Kappa = 0.3573, 95% CI = 0.2282–0.4864). However, the symmetry test did not reject the hypothesis of equality between the two sides (*p* value = 0.4040).

No significant differences were found between Keros scores and age regarding the right (*p* value = 0.4620; ANOVA) and left sides (*p* value = 0.5709), as shown in Table 2.

Table 3 shows that there is also no association between Keros scores and gender on the right (*p* value = 0.1421, chi-square test) and left (*p* value = 0.2136, chi-square test) sides.

In addition to the analysis for each side, the comparison between genders and ages was carried out considering both sides simultaneously (GEE). In this analysis, neither significant difference was observed between sides nor between genders or between ages, as shown in Table 4. Furthermore, there were no significant interactions between side and gender or between side and age.

## 4. Discussion

Endoscopic management of the paranasal sinuses raises issues on possible risks of perforation during the surgical procedure, with emphasis on regions of the ethmoidal roof and cribriform plate corresponding to the olfactory fossa because of their inherent characteristics of fragility resulting from their extremely thin composition [14]. Başak et al. [18] pointed out the importance of the previous studies prior to interventions in the paranasal sinuses for identification of anatomical variations, including localization of the anterior ethmoidal artery and cribriform plate of the ethmoidal bone. Among the different modalities of imaging examinations for this aim, one can highlight the CT as this modality allows identification of anatomical changes in the region of the rhinosinusal complex and location of vital structures. Studies in the final of the 1990s culminated in a new method of image acquisition called CBCT for the facial region, allowing individualized sections with submillimetric thickness to be acquired, which provides high-resolution spatial images of bone tissues.

CBCT are reported to be excellent tool for visualization of the inner structures in the skull base, with high spatial resolution and contrast variation among tissues [19, 20], allowing the anatomical position of target organs [21].

This methodology has the advantage of significantly reducing the radiation dosage to the patient compared to MSCT. For instance, one can cite the radiation dose of a MSCT examination for a dental arch, which is, on average, 1.5 to 12.3 times higher than that of CBCT [22].

In this context, the present study has characterized the variations in olfactory fossa considering possible relationships of dimensional variations, side, age group, and gender by means of CBCT images. Therefore, the Keros classification [7] was used. Its importance is based on the objective of establishing a possible morphological pattern in order to assist local surgical approaches in preventing injuries to associated structures.

With regard to the frequency of Keros types, type II was the most often observed in our study. This finding corroborated those reported elsewhere [14, 17, 23–26]. We have found in the literature only one study [27], where type I was more frequent than type II, but it should be emphasized that their sample was the smallest (i.e., 50 MSCT examinations) among all the studies reviewed and also presented a prevalence of children with no balanced age distribution, which might have influenced the results. It should be also emphasized that our study used a sample of 174 CBCT examinations, which was quantitatively expressive compared to other studies, in order to investigate types of olfactory based on the Keros classification. On average, 100 MSCT examinations were assessed [23, 28], except a study by Güldner et al. [14] in which a sample of 865 examinations was used.

No statistically significant differences between Keros types for olfactory fossa on the right and left sides were found in the present study (Table 1). On the contrary, there are studies reporting a significant difference between the sides [17], who found a higher trend of extreme values compared to the Keros classification, being either extremely shallow or intensively deep, as well as the study by Erdogan et al. [25], who also reported a difference between the sides. A study investigated a specific Malaysian population and also found a deeper olfactory fossa on the left side [23]. However, the present work corroborates the results reported by several previous studies regarding the sides and which were based on MSCT images, demonstrating that CBCT supports the most frequent findings regarding this modality and is a reliable alternative, with advantages over the former regarding side effects to the patient (e.g., reduced radiation dose).

Our study has revealed no statistical differences regarding Keros types and age group (Table 2) on both sides. In a previous study [14], the authors assessed scans of patients from two groups (i.e., those younger than 18 and those older than 18 years) and observed a higher frequency of Keros type I in the former; that is, their olfactory fossa was shallower, whereas in the latter, it was deeper (higher frequency of Keros type II). To some extent, we consider that our findings are in accordance with their results as our sample consisted of CBCT examinations acquired from adult patients (i.e., older than 18 years), with Keros type II being the most frequently observed.

It is important to emphasize that the study by Güldner et al. [14] was the only one found in the literature using CBCT images, like ours, rather than MSCT ones, like others. However, the differential is that their objective was to globally assess anatomical elements of the skull base in relation to the age group, but without assessing the distribution of Keros types in relation to gender and side.

The effective dose of CBCT varies according to the different CBCT scanners and with the technical specifications selected during the scan: milliampere, kilovoltage, FOV, and exposure time [19, 29]. However, the dose is significantly reduced compared to MSCT [19, 20].

This methodology has the advantage of significantly reducing the radiation dosage to the patient compared to MSCT. For instance, one can cite the radiation dose of a MSCT examination for a dental arch, which is, on average, 1.5 to 12.3 times higher than that of CBCT [22].

Previous study has shown the correlations between MSCT and CBCT about the FOV size for imaging of sinus and middle ear [30]. Even in larger FOVs, the doses for CBCT sinus and middle ear are lower than for MSCT; therefore, it can be justified to replace some MSCT examinations by CBCT examinations [30].

In the present study, the distribution of the different Keros types was not differentiated by gender, despite the considerable proportion difference in the sample between number of CBCT examinations belonging to females (*n* = 143; 82.2%) and males (*n* = 31; 17.8%). Muñoz-Leija et al. [26] also found a gender difference, being the only study to obtain this result regarding this variable. Although it might be supposed that male gender could influence the depth of the olfactory fossa, resulting in more pronounced skeletal dimensions and consequently in a greater tendency of Keros type III, we believe that there is a proportionality in the overall growth of the components of the olfactory fossa complex that results in a structure undergoing no variation due to sexual dysmorphism.

The possible simultaneous influence of both gender and age on the distribution of Keros classification was not demonstrated in our study sample, regardless of the sides investigated (i.e., right and left; *p* > 0.005) (Table 4). Therefore, according to the classification used, there is evidence that morphological aspects of the olfactory fossa are not influenced by gender, side, or age, despite a higher frequency of Keros type II.

The overall concordance between our findings and those reported by previous studies using MSCT shows that CBCT can be alternatively used for treatment planning and local manoeuvres (e.g., endoscopic procedures in the ethmoidal region). The use of CBCT images, even with lower spatial resolution, for describing the olfactory fossa becomes important as otolaryngology specialists are increasingly requesting CBCT examinations to evaluate paranasal sinuses and surrounding structures based on its properties.

## 5. Conclusions

This study shows no significant association with Keros classification, gender, and age in our sample. CBCT was a useful tool for evaluation of the anterior skull base.

## Figures and Tables

**Table 1:** The distribution of Keros classification and the measures according to sides.

Right	Left			
	I	II	III	Total
I	**14**	10	0	
	**8.05** ^1^	5.75	0.00	24
	**58.33** ^2^	41.67	0.00	13.79
	**63.64** ^3^	9.17	0.00	

II	7	**80**	27	
	4.02	**45.98**	15.52	114
	6.14	**70.18**	23.68	65.52
	31.82	**73.39**	62.79	

III	1	19	**16**	
	0.57	10.92	**9.20**	36
	2.78	52.78	**44.44**	20.69
	4.55	17.43	**37.21**	

Total	22	109	43	174
	12.64	62.64	24.71	100.00

^1^Percentage of the total subjects (174 subjects); ^2^percentage of the row (sum 100% on the row); ^3^percentage of the column (sum 100% on the column).

**Table 2:** Measures of position and dispersion of age by the Keros classification (ANOVA).

Side	Keros	*N*	Mean values	SD	*p* value
Right	I	24	47.75	11.80	0.4620
	II	114	45.37	12.11	
	III	36	43.61	14.61	

Left	I	22	44.86	14.39	0.5709
	II	109	46.07	12.91	
	III	43	43.70	10.85	

*N*: number; SD: standard deviation; *p* value significant at 5%.

**Table 3:** Distribution of the Keros classification according to gender.

Side	Gender	Keros I		Keros II		Keros III		*p* value
		*N*	%	*n*	%	*n*	%	
Right	F	23	95.83	90	78.95	30	83.33	0.1421
	M	1	4.17	24	21.05	6	16.67	

Left	F	21	95.45	88	80.73	34	79.07	0.2136
	M	1	4.55	21	19.27	9	20.93	

*N*: number; F: female; M: male; *p* value significant at 5%.

**Table 4:** GEE results to assess the influence of gender and age on the Keros classification.

Gender		Age	
Variable	*p* value	Variable	*p* value
Gender	0.1300	Age	0.2367
Side	0.1931	Side	0.8465
Side ∗ gender	0.6190	Age ∗ side	0.6344

## Data Availability

The data used to support the findings of this study are available from the corresponding author upon request.
